# The Relationship between Socioeconomic Status, Mental Health, and Need for Long-Term Services and Supports among the Chinese Elderly in Shandong Province—A Cross-Sectional Study

**DOI:** 10.3390/ijerph16040526

**Published:** 2019-02-13

**Authors:** Fanlei Kong, Lingzhong Xu, Mei Kong, Shixue Li, Chengchao Zhou, Jiajia Li, Long Sun, Wenzhe Qin

**Affiliations:** 1 Key Lab of Health Economics and Policy Research, School of Public Health, Shandong University, 44 Wenhuaxi Road, Jinan 250012, China; kongfanlei@msn.com (F.K.); shixueli@sdu.edu.cn (S.L.); zhouchengchao@sdu.edu.cn (C.Z.); lijiajia@sdu.edu.cn (J.L.); sunlong@sdu.edu.cn (L.S.); qinwenzhe09@163.com (W.Q.); 2 Collaborative Innovation Center of Social Risks Governance in Health, School of Public health, Fudan University, Shanghai 200032, China; 3 Research Center of Economics and Resource Management, Beijing Normal University, Beijing 100875, China; kongmei007@126.com

**Keywords:** socioeconomic status, mental health, need for long-term services and support, Chinese elderly, structural equation modeling

## Abstract

This study aims to clarify the association between socioeconomic status (SES), mental health, and the need for long-term services and support (NLTSS) of the Chinese elderly, and further, to provide evidence-based suggestions for the development of the long-term services and support (LTSS) system in China. A cross-sectional survey using a multi-stage random sampling method was conducted in Shandong Province, China, in 2017. Data were collected from seniors aged over 60 years old through questionnaires by face-to face interviews. A total of 7070 subjects were included in the final database (40.3% male and 59.7% female). A chi-square test analysis and structural equation modeling (SEM) were employed to explore the relationship between SES, mental health, and NLTSS for both male and female elderly people. The SEM analysis showed that mental health was significantly and negatively associated with NLTSS for both male elderly and female elderly, and it was slightly stronger among the male elderly. A significant and negative relationship was observed between SES and NLTSS for both genders, and the association was stronger among the female elderly. SES exerted a positive effect on mental health for both male and female elderly people, and a slightly stronger effect was found among the male elderly. Advice for the development of a LTSS system in China was given based on the above results.

## 1. Background

With the decline in the fertility rate and increase of life expectancy, population aging—the inevitable increase of the percentage of older adults—is occurring throughout the world. The global population aged 60 years or over numbered 962 million in 2017, more than twice as large as in 1980 when there were 382 million older persons worldwide. This means that older people comprised 13% of the global population in 2017 [1]. The population aged 60 and above is growing at a rate of about 3 percent per year, and all regions of the world (except Africa) will have nearly a quarter or more of their populations at age 60 and above by 2050. The number of older persons in the world is projected to be 1.4 billion in 2030 and 2.1 billion in 2050, and could rise to 3.1 billion in 2100 [2]. It is worth noting that two thirds of the world’s elderly live in developing regions, where their number is growing faster than in developed regions. In 2050, it is expected that nearly 8 in 10 of the world’s older persons will be living in developing regions [2]. China, as the biggest developing country in the world, has also experienced rapid population aging in the past decades. At the end of 2017, there were 241 million older adults aged more than 60 years old in China, accounting for 17.3% of the total Chinese population. It is projected that there will be 487 million elderly people in China, constituting 34.9% of the whole population, in 2050 [3]. Ten million new elderly emerged in 2017, which was more than twice the total population of the Philippines, and more than four times the total population of South Africa [4].

Many researchers have clarified the relationship between socioeconomic status (SES) and mental health in different regions. For example, adults living in high-income inequality counties have been found to have worse mental health than those in low-income inequality counties [5], and the negative impacts of displacement on mental health were found among adults living in urban areas [6], which showed SES was associated with mental health [7]. Concerning the Japanese elderly, the prevalence of poor mental health became lower as SES became higher [8], indicating the positive relationship between SES and mental health for both genders of Japanese elderly [9]. What is more, low childhood SES in post–World War II has had a long-latency effect on the onset of depression among Japanese adults [10]. Health disparities by SES exist for many outcomes, and generally speaking, people with low SES are more likely to experience a lower subjective health status after myocardial infarction [11], higher rates of psychiatric morbidity and use of psychiatric services [12], and increased risk of suicidal ideation among the elderly [13].

Previous studies have shown that the elderly in lower income brackets have worse functional impairment than those who reported higher levels of income [14], and low-income single people were more likely to use home care among the single-living older people [15], which illustrates that SES is generally negatively associated with the need for long-term services and support among the elderly [9]. Low SES not only increased the risk for entering LTSS [16], but also increased the difficulties in accessing formal care [17]. Specifically, the effects of socioeconomic factors on entry into LTSS were stronger among men than women [16]. What is more, SES is an important factor influencing preferences for long-term care (LTC) arrangements among the elderly [18]. Concerning the NLTSS among minority groups, Tennstedt and Chang’s study illuminated the important role played by ethnicity in explaining the elderly’s need for and receipt of LTSS assistance, since elders in minority groups received more informal care than older white persons [19]. On the other hand, Miller’s research showed few differences in change patterns of community long-term care among African American and white frail older persons [20].

Concerning the association between mental health and NLTSS, perceived need for psychological/psychiatric services was quite high among LTSS patients with diabetes in LTSS facilities [21], and increased utilization of medical services among elderly patients with depression was found in primary care practices [22]. The provision of optimal mental health care services in LTSS facilities is dependent on adequate funding, availability of expertise and education, positive and caring attitudes, recognition of needs, and supportive teamwork [23]. Research with Japanese elderly showed that mental health exerts a negative effect on NLTSS, and the effect was stronger for Japanese elderly women [9]. Additionally, a small social support network, heart disease, and daytime sleepiness were associated with low mental health well-being for those with NLTSS insurance [24]. It is well-known that the caregiver plays an important role in the NLTSS system, including in health promotion programs, as their encouragement towards participation is effective for improving physical and mental health outcomes among residents in long-term care facilities [25]. However, it is worth noting that mental health problems and emotional strain increased significantly over time among professional caregivers [26].

Some previous studies have explored the relationship between two variables, such as the association between SES and mental health, between SES and NLTSS, and between mental health and NLTSS, yet no research has ever clarified the relationship between SES, mental health, and NLTSS by using Structural Equation Modeling (SEM) among the Chinese elderly in a traditional majority Chinese population living area. This study aimed to address this research gap, and additionally to determine whether there is gender difference in the association between SES, mental health, and NLTSS among Chinese elderly in Shandong Province.

## 2. Methods

### 2.1. Study Location and its Population Aging Conditions

Shandong Province is a coastal province of China which lies between Beijing and Shanghai, belonging to the Eastern China region. Within the 15.79 km^2^ administrative region (420 km north to south, 700 km east to west), there are 17 prefecture-level cities, 137 counties, and 1826 towns. Shandong Province has been very important in Chinese history since the beginning of Chinese civilization along the Yellow River, and has served as a pivotal cultural and religious site for Taoism, Chinese Buddhism, and Confucianism [27]. The Gross Domestic Product (GDP) of Shandong Province in 2017 was 7267.82 billion RMB (1076.40 billion U.S. dollars), increasing 7.40% over the previous year. GDP-per-capita was 72,851 RMB in 2017, equal to 10,790 U.S. dollars. The resident population in Shandong Province was 100,058.30 thousand people at the time this research was carried out, of whom 13.99% were elderly people older than 65 years in age [28].

### 2.2. Data Collection and Research Participants

The data was collected by face-to-face interviews using a structured questionnaire in the 2017 Survey of the Shandong Elderly Family Health Service, which was conducted by Shandong University. A multi-stage random sampling method was employed to select the participants following the three steps below. Firstly, six counties were selected from 137 counties as the primary sampling units (PSUs) throughout the eastern, central, and western regions of Shandong Province (which were divided into three districts and three counties that represented urban and rural areas, respectively). Secondly, 18 villages in rural areas and 18 communities in urban and suburban areas were selected from each PSU as the secondary sampling units (SSUs). Thirdly, based on the roster of the residents by age and the total elderly population of each selected site provided by the local residential committee, an average of 66 individuals were stratified and randomly selected from each SSU, making up the total sample. A 30-minute face-to-face interview was conducted in every research participant’s home by a Doctoral or Master’s student of Shandong University.

The eligible participants included in the survey were those age 60 or older, with local household registrations at the time of the interview. A total of 7088 elderly individuals were initially selected and interviewed. Of these, 18 were excluded from the sample because of an uncompleted questionnaire or an obvious logical error in the questionnaire. Finally, a total of 7070 individuals were included in the database.

### 2.3. Measurements

All of the definitions and measurements (and the corresponding options) of the variables are included in Table 1. SES is a broad concept that compositely assesses an individual’s economic and sociological position in relation to others, and mainly includes factors such as education level, occupation, income, wealth, and deprivation [29,30]. In this study, education, annual income and job before retirement were considered as measures of SES. In 1973, Kitagawa and Hauser used educational attainment as the main indicator of SES [31]. Since then, education has played a central role in the SES-health gradient analyses. Education not only shapes a person’s the future employment opportunities and earning potential, but also provides knowledge and life skills that enable better-educated people to access information and resources to promote health, so it could be seen as the most basic SES component [32]. In this study, education was measured by asking the subjects the question as “which level of education did you finish?” with four choices: (1) Illiteracy, (2) Graduate from Elementary School, (3) Graduate from Junior Middle School and (4) Graduate from Senior Middle School and above. Researchers have found that low income is associated with many other factors which contribute to poor health, including risky health behaviors, lower education levels, food insecurity, unqualified housing, and lack of health insurance coverage. Even if most other factors are controlled, income also may be independently related to health outcomes [33]. Researchers have even regarded income as ‘one of the most profound influences on mortality’ [34]. The annual income was measured by the following categories in this study: (1) less than 2200 RMB (USD = 326), (2) from 2201 to 3300 RMB (USD = 489), (3) from 3301 to 6200 RMB (USD = 919), (4) from 6201 to 21,400 RMB (USD = 3170) and (5) more than 21,400 RMB (USD = 3170). Occupational status, such as insecure jobs [35], unemployment [36], high job strain [37], and a high level of occupational physical activity [38] were found to be risk factors for health. In consideration of the effect of their job before retirement on the social life and health conditions of the sampled elderly people after retirement, job before retirement was included in SES in this study. It was measured by the following options: (1) Professionals/technical, (2) Leaders of government/public institution, (3) Clerks or staff, (4) Businessman/commercial servants, (5) Farming/forestry/fishing industry, (6) Transportation industry/manual workers, (7) Privately/individually-owned business, and (8) Other jobs.

The World Health Organization (WHO) has defined mental health as a state of well-being in which every individual realizes his or her own potential, can cope with the normal stresses of life, can work productively and fruitfully, and is able to make a contribution to her or his community [39]. In this study, mental health was assessed by three questions: (1) “How do you feel about your health status (subjective health)?” (2) “How was your mental health status last month (mental health last month)?”, and (3) “Are you satisfied with your life (life satisfaction)?”.

So far, there is no national long-term care insurance (LTCI) system in China. We used (1) Do you need long-term care, and (2) Activities of Daily Living (ADL) score to evaluate the NLTSS of the participants in our analysis. The ADL scale consists of the Physical Self-Maintenance Scale (PSMS) and the Instrumental Activities of Daily Living Scale (IADL) [40], which are mainly used to assess the daily living ability of the subjects by totaling the scores from 14 items. The six items of the PSMS include going to the toilet, eating, dressing, grooming, walking, and bathing. The eight items of the IADL include telephoning, shopping, food preparation, housekeeping, laundering, using transportation, taking medicine, and financial behavior. During the survey, the interviewer circled the most suitable of the choices (1: can do it by myself, 2: is difficult, 3: need help, 4: can’t do it), according to the observation of subjects and their reply. If the subject could not answer or could not answer correctly (such as in the case of subjects with dementia or aphasia), it could be assessed according to the observation of family members, nursing staff, and other insiders. The total score is at least 14 points when it is completed normally; >14 points indicates some functional decline, and the highest possible score is 56. When two or more items ≥3 points, or a total score is obtained of ≥22 points, it means there is obvious dysfunction.

### 2.4. Statistical Analysis

Descriptive statistics were employed to describe the participants’ socio-demographic characteristics. A chi-square test was used to determine the gender difference in socio-demographic characteristics, SES indicators, mental health indicators and NLTSS indicators separately. *p* Value less than 0.001 were considered to be statistically significant, and all the analyses above were carried out by using the Statistical Package for Social Science for Windows (SPSS, version 25.0, IBM, Armonk, New York, USA).

Structural Equation Modeling (SEM) was employed to explore the association between the SES, mental health and NLTSS of the Chinese elderly in Shandong Province. Maximum-likelihood estimation is used to estimate the best-fitting model in this study. The SEM model consists of two types of variables: exogenous variable and endogenous variable. The exogenous variable in this study was SES, and the endogenous variables were mental health and NLTSS. The short introduction of SEM can be seen in Appendix A. The present study used AMOS (version 25.0, IBM, Armonk, New York, USA) statistical software package for Windows to run SEM to obtain maximum-likelihood estimates of model parameters and calculate the model fitness indexes.

### 2.5. Ethical Considerations

This study was approved by the ethical committee of Shandong University (No. 20170110). Informed consent for the data collection and use of information was obtained from all participants.

## 3. Results

### 3.1. Sample Characteristics

The distribution of socio-demographic characteristics is summarized in Table 2. In total, 7070 participants were included in our analysis, of whom 2846 (40.3%) were male, and slightly less were females (4224, 59.7%). As for long-term services and supports needs (LTSSN), there were more females both in the ‘Not Needed’ group (4046 for females and 2722 for males) and ‘Needed’ group (178 for female and 124 for male). No statistical difference between the male elderly and female elderly on LTSSN was found. There were 302 elderly who needed LTSS, accounting for 4.3% of Chinese elderly in the current study. With respect to age, 1577, 2129, 1780 and 1584 participants belonged to the age groups of 60–64, 65–69, 70–74, and >75, respectively. From these age groups, 57, 69, 69, and 107 needed LTSS, indicating the elderly in higher age groups needed more LTSS. A statistical difference between the different age groups on LTSSN was found.

As demonstrated in Table 3, 2924 of the participants had an education level of elementary school, followed by illiteracy (2270), junior middle school (1315), and senior middle school or above (561). In terms of annual income, 1685 of the elderly belonged to the 0–2200 group, followed by the >21,400 group (1414), 6210–21,400 group (1414), 3301–6200 group (1404), and 2201–3300 group (1153). A total of 5479 of the participants’ jobs before retirement was farming/forestry/fishing, followed by professional/technical (442), transportation industry/manual worker (383), other jobs (274), leaders of government/public institution (180), clerks/staff (164), merchant/commercial servants (116), and private/individually-owned business (32). A statistical difference in education, annual income, and job before retirement between male elderly and female elderly participants was observed.

As illustrated in Table 4, 2639 of the participants rated their health status as “fairly good”, followed by “moderately good” (1992), “fairly bad” (1169), “very good” (1143), and “very bad” (127). As for life satisfaction, 3939 of the subjects were definitely satisfied with their life, followed by “very satisfied” (2822), “moderately satisfied” (135), “fairly dissatisfied” (88) and “very dissatisfied” (87). A total of 3361 of the participants thought their mental health in last month was good, followed by “very good” (2190), “moderately good” (1192), “bad” (288), and “very bad” (39). Statistical differences in subjective health and mental health in the last month were found between male and female participants, but no statistical difference was found in life satisfaction.

Of the 7070 participants, 5467 had an ADL score of 14, followed by 18–27 (525), 15 (482), 16 (222), 17 (205), and ≥28 (169). A statistical difference in ADL score between male and female participants was found (as shown in Table 4).

### 3.2. The Structural Model

#### 3.2.1. Measurement Invariance Across Gender

Table 5 illustrated the results about the related fit statistics of the measurement invariance across gender and the fitness indexes in five selected models. In order to check whether the variable ‘gender’ is suitable for the group comparison, the fitness indexes between the male elderly and female elderly should be compared first.

The model fitness indices used in the current study were GFI (goodness of fit index), AGFI (adjusted goodness of fit index), CFI (comparative fit index), RMSEA (root mean square error of approximation), the corresponding standard for each index was shown in Appendix B. As is shown in the table, the fitness indexes of the male elderly and female elderly were the same (GFI = 0.993, AGFI = 0.983, CFI = 0.975, RMSEA = 0.029 are same in M_1_ and M_2_), implying that we could furtherly check the measurement invariance between the male elderly and female elderly on the other following models.

Then we use the ΔCFI and ΔRMSEA between M3 (Unconstrained Model), M4 (Measurement Weights Model) and M5 (Structural Weights Model) to evaluate the measurement invariance. The M3 does not restrict any coefficient in the model, the M4 assumes the indicator loadings for the corresponding construct of each group are equal, while the M5 constrains the indicator loadings of the corresponding construct and the structural coefficients between the groups.

As seen from Table 5, the ΔCFI between M4 and M3 is 0.009, and between M5 and M4 is 0.001. All of the ΔCFI values were less than 0.010, indicating the measurement invariance was established between the models of M1, M2, M3, M4 and M5 across gender (the principles can be seen in Appendix C). The ΔRMSEA between M4 and M3 is 0.002, and between M5 and M4 is 0. All of the ΔRMSEA values were less than 0.015, also indicating the measurement invariance was established between the models of M1, M2, M3, M4 and M5 across the group of male elderly and female elderly (the principles can be seen in Appendix C).

#### 3.2.2. Model Fitness Indices

**Figure 1:**
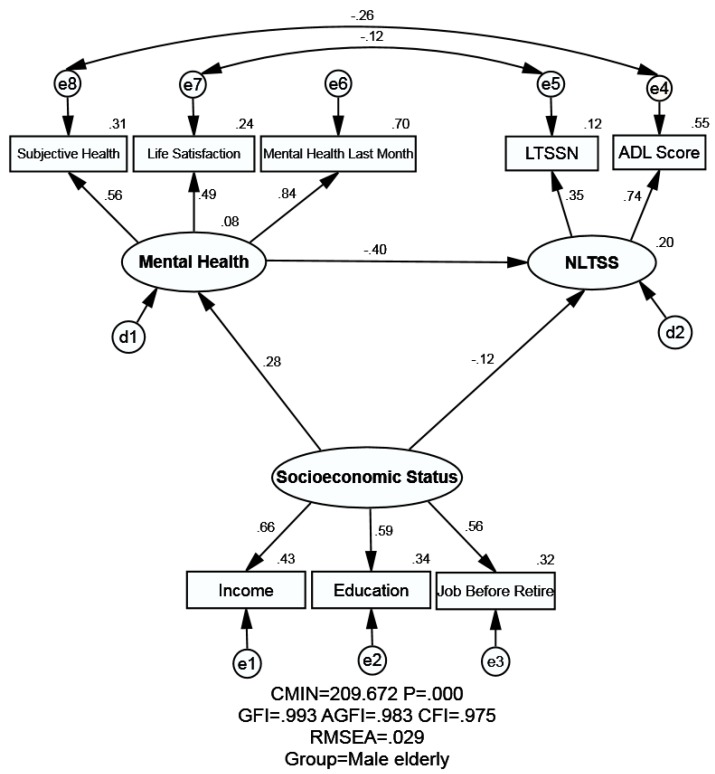
Structural equation modeling analysis of the association between SES, mental health and NLTSS of the male elderly (*n* = 7070). Employing the cross-sectional data, relationship between SES, mental health and NLTSS were analyzed. Arrows indicate the associations and directions between variables, double curved arrows indicate correlation between each factor. All parameter estimates were statistically significant (*p* < 0.001). Note: *χ^2^* = Chi square; GFI= Goodness of Fit Index; AGFI = Adjusted Goodness of Fit Index; CFI = Comparative Fitness Index; RMSEA=Root-mean Square Error of Approximation; LTSSN = Long-term Services and Supports needs; SES = Socioeconomic Status; NLTSS = Need for Long-term Services and Supports.

**Figure 2:**
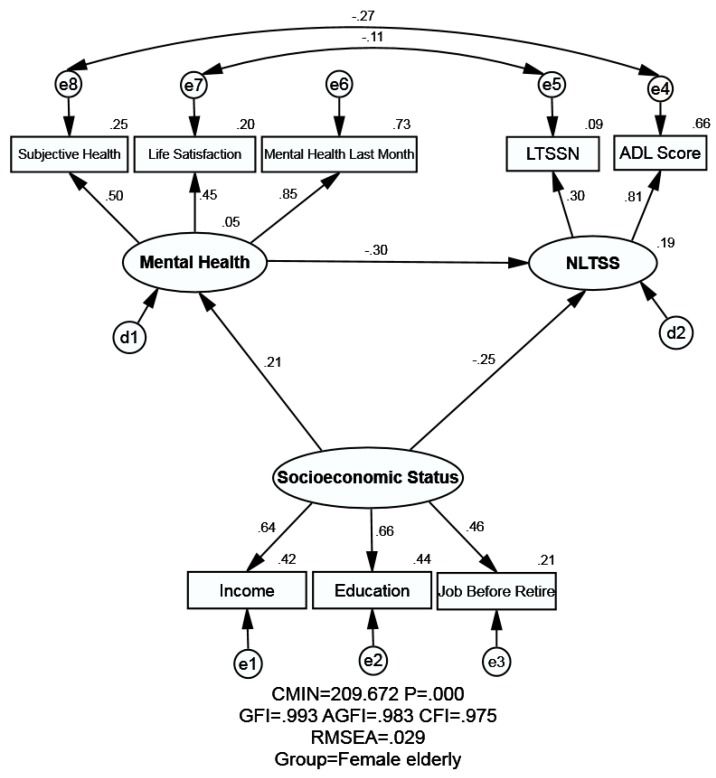
Structural equation modeling analysis of the association between SES, mental health and NLTSS of the female elderly (*n* = 7070). Employing the cross-sectional data, relationship between SES, mental health and NLTSS were analyzed. Arrows indicate the associations and directions between variables, double curved arrows indicate correlation between each factor. All parameter estimates were statistically significant (*p* < 0.001). Note: *χ^2^* = Chi square; GFI = Goodness of Fit Index; AGFI=Adjusted Goodness of Fit Index; CFI = Comparative Fitness Index; RMSEA = Root-mean Square Error of Approximation; LTSSN = Long-term Services and Supports needs; SES = Socioeconomic Status; NLTSS = Need for Long-term Services and Supports.

The proposed models of the male elderly and the female elderly are illustrated by Figure 1 and Figure 2 respectively, which contain three latent variables: SES, mental health and NLTC. Table 5 shows the model fitness indexes in different model, yet here we just focus on M_1_ (male elderly) and M_2_ (female elderly). The estimated value of model fitness for the male elderly and female elderly were same: GFI = 0.993 > 0.90, AGFI = 0.983 > 0.90, CFI = 0.975 > 0.90 and RMSEA = 0.029 < 0.05, indicating that the theoretical model fit the empirical data very well for both the male elderly and female elderly (the principles can be seen in Appendix C). The Chi-square value in Amos is called CMIN, and it was significant as seen in Table 5 (*p* < 0.001). Yet the CMIN was not employed to evaluate the fitness in this study like many other researchers due to it is sensitive to the samples size.

### 3.3. Relationship between SES, Mental Health and NLTSS Assessed with SEM

The association between SES, mental health, and NLTSS is shown in Table 6, Figure 1, and Figure 2. SEM could analyze not only the empirical relationship among different variables in the model, but also the statistical association between the observed variables and unobserved variables simultaneously. In this study, there are three unobserved variables: SES, mental health, and NLTSS. SES is measured by three observed variables, namely education, income, and job before retirement. Mental health is measured by three observed variables: subjective health, mental health in the last month, and life satisfaction. NLTSS is measured by two observed variables: LTSSN and ADL score.

#### 3.3.1. Association between SES and NLTSS

SES and its indicators exerted the effect on NLTSS both directly and indirectly (through mental health). The negative and direct effect from SES on NLTSS was observed both for male (standardized direct effects = −0.117) and female elderly (standardized direct effects = −0.252). Meanwhile, SES could affect NLTSS negatively and indirectly via mental health (standardized indirect effect of −0.113 for male elderly and −0.064 for female elderly). Eventually, SES exerted a stronger negative effect on NLTSS for female elderly than male (standardized total effects = −0.316 for female elderly; standardized total effects = −0.230 for male elderly), which meant the higher SES groups would generally have less NLTSS. It was worth noting that the standardized total effects of both groups were statistically significant, yet very little difference was observed between male elderly and female elderly.

#### 3.3.2. Association between Mental Health and NLTSS

A negative and direct relationship was observed between mental health and NLTSS among male and female elderly, which meant that a better status of mental health of the elderly would indicate lower NLTSS. Statistical significance was found across genders, and the coefficient was slightly stronger among male elderly (standardized total effects = −0.400 for male elderly; standardized total effects = −0.300 for female elderly).

#### 3.3.3. Association between SES and Mental Health

As for the relationship between SES and mental health, a positive and direct effect was found both for the male elderly and female elderly (standardized total effect = 0.281 for male elderly; standardized total effect = 0.214 for female elderly), indicating the higher the SES of the elderly, the better their mental health. Statistical significance was found across genders, yet the relationship between SES and mental health was higher for male elderly than female elderly.

## 4. Discussion

### 4.1. Principal Findings and Comparison with Other Studies

Employing SEM, this study investigated the relationship between SES, mental health, and NLTSS. The results revealed that both SES and mental health affect NLTSS, and SES was also correlated with mental health. A slight gender difference was also found for the above three associations.

#### 4.1.1. NLTSS of the Participants

The majority of older adults in this study did not need long-term services and support, and this result is similar to a study conducted among Japanese elderly in Tokyo [9]. Another research study carried out among elderly people in Vietnam, with the purpose of assessing their required support in ADLs, also found that most of the participants did not need any support [41]. It is worth noting that both China and Vietnam have not established a national LTCI system currently, while Japan founded their LTCI system in the year 2000. Although China, Vietnam, and Japan are in different development stages and are implementing different health care policies (in particular in terms of whether LTCI is established or not), that the majority of elderly in these three studies do not need LTSS may indicate the good health conditions of the general population in the research locations. What is more, the similar results between these three studies also showed the comparability between self-reported LTSSN by elderly from those countries that have not established a national LTCI (such as China), and the officially functionally assessed LTSSN from those countries that have established a national LTCI (such as Japan).

#### 4.1.2. SES and NLTSS

The negative correlation between SES and NLTSS, clarified in the present study with Chinese male and female elderly, was similar to the findings of previous studies with the Japanese elderly population [9] and the elderly in Vietnam [41], which also showed socioeconomic factors (educational attainment, equivalent income, etc.) were related to the NLTSS of the elderly. This result may explain why previous studies have found that higher levels of unmet needs were reported by people living in the most deprived areas [42], and why a lower SES tended to indicate the increase of the propensity and intensity of care utilization [43]. Concerning the gender differences, the results of the present study showed SES indicators exert a slightly stronger effect on NLTSS for female elderly than male. This was different from the findings of a study conducted in Finland, which indicated that the effect of SES factors on LTSS was stronger among male elderly [16].

#### 4.1.3. Mental Health and NLTSS

The negative correlation between mental health and NLTSS among Chinese elderly in this study was the same as found in the study conducted with Japanese elderly [9]. Most of the previous studies explored the prevalence of NLTSS among frail people or patients, yet few have clarified the empirical association between mental health and NLTSS. The elderly with dementia [44], intellectual disabilities [45], and stroke survivors [46] had never been specifically studied in this area; although the NLTSS of these groups has been highlighted, the empirical correlation between mental health and NLTSS was not demonstrated. This study went further than previous researchers have, in order to verify the empirical relationship between mental health and NLTSS. The empirical association illustrated in this study also explained why 53% of people with dementia eventually received both home help and institutional care, while 34% and 12% of people with other psychiatric diagnoses and people with good mental health, respectively, eventually receive this type of care [47].

#### 4.1.4. SES and Mental Health

The positive correlation between SES and mental health found in this research with Chinese elderly was the same as in the study among Japanese elderly [9]. This result may also explain why the population whose SES was lower tended to have more mental health problems [48], while people with higher SES usually have better mental health [8,49,50]. Concerning the gender differences in the association between SES and mental health, a stronger correlation was found among female elderly than male elderly. This was different from the previous study, which found that educational attainment was statistically significantly correlated with mental health for Japanese female elderly, but no association was found for Japanese male elderly [51]. This might because of the different cultural backgrounds in China and Japan, as well as the different compositions of research participants.

#### 4.1.5. SES, Health and NLTSS

Numerous studies have explored the association between health and SES, and a positive association between global health status and SES has consistently been found, implying a higher SES would generally come along with a better health status [30,52,53,54]. The health status of the population from different levels of SES was influenced by lifestyle, environmental, and social factors [55]. Ecological factors were also found to be mediators in the relationship between SES and health status [56]. To be specific, the disparity of SES could also imply a different level of access to health services, exposure to occupational hazards and environmental pathogens, low levels of social support and social capital, poor social policy, the cumulative effects of stress, and differences in health risk behaviors [57].

Higher and higher life expectancies, more and more fragile pension and social security systems around the world, and less and less extended families and traditional systems of support for the elderly have all made the concerns regarding the relationship between SES and health among older adults particularly important [58]. In this study, we clarified the relationship between SES, mental health, and NLTSS using SEM, and identified the empirical correlations among Chinese elderly. According to the results of the previous research, the findings of this study could be explained as follows: seniors from different SES levels live in different communities with different environments, live a different daily lifestyle, and have a different level of access to health services. These factors lead to different mental health outcomes among the elderly. As the mental health of the elderly becomes worse, their NLTSS gradually emerges and increases.

Compared with the study conducted among the Japanese elderly, two important differences were observed. Firstly, although the direction of all the correlation coefficients (positive/negative) were same, the absolute value of the correlation coefficients in the SEM were higher among the Chinese elderly than Japanese elderly in the current study. The second difference is that the association between mental health and NLTSS is statistically significant and negative for both Japanese and Chinese elderly for both genders, but it was stronger among Japanese female elderly than Japanese male elderly, while it was stronger among Chinese male elderly than Chinese female elderly. These differences may imply that there is a gap in economic and social development between China and Japan. Although China is ranked second in the world by GDP, owing to the huge population, the GDP-per-capita (in current U.S. dollars per person) of China was about only 20% of Japan and 14% of United States in 2016 [59]. In the current development stage, the value and importance of income (an important indicator for SES) for Chinese people and Japanese people are at different levels. China, as the biggest developing country, is still on her way to development, yet Japan had been the most developed country in Asia for decades. This may lead to the value of SES related indicators (especially income) being comparatively higher among the Chinese people than the Japanese people. In the present study, SES was the basic independent variable to affect mental health and NLTSS. It might because of these factors that the absolute values of the correlation coefficients in the SEM were all higher among the Chinese elderly than Japanese elderly in the current study. As for the gender differences in the association between mental health and NLTSS, they may have occurred because of the different characteristics of the research participants, or the different cultural backgrounds on gender inequality.

### 4.2. Implications

China has developed a mixed social security pension system with a defined benefit pay-as-you-go portion, and an investment-based defined contribution portion [60]. In terms of health insurance, after continuous reforms in the past decades, China’s basic social medical insurance system has been developed to include the Urban Employee Basic Medical Insurance system (UEBMI, for those who have jobs in the city), the Urban Resident Basic Medical Insurance system (URBMI, for those who have no job in the city) and the New Rural Cooperative Medical System (NRCMS, for those who live in the city) [61]. These three kinds of medical insurance cover the majority of the Chinese people, and a small proportion of Chinese purchased commercial medical insurance [62]. However, a national LTCI system, which could not only provide caring services and support for the elderly and their family, but also lower the out-of-pocket payment for caring, has not been established in China so far. As more and more countries enter economic and social development influenced by having an aged society, evidence-based age friendly policies are increasingly needed, and the construction of an LTSS system is one of these imperative policies. The results of the current study on the empirical relationship between SES, mental health and NLTSS could be useful in the development of an LTSS system, for both China and other developing countries, which will play a very important role for aging populations.

Firstly, the fundamental role of SES in mental health and NLTSS implies that social and political initiatives are needed to enhance the SES of elderly people, especially in the field of social security and welfare, tax-and-transfer, and the labor market. This cannot be easily achieved through interventions on the individual level, however public policy provides early intervention and prevention for the vulnerable elderly and their families, which may advance their SES and lower the SES inequality in the long run. To be specific, the government needs to implement the related policies on increasing the income, education level, and social status of older adults, as well as to ensure medical coverage for mental health and long-term services and support for all older adults, regardless of their ability to pay for access to health care [62]. For example, income levels could be increased by raising the pension fee through the redistributive system, or lowering the expenditure on LTSS. As for educational attainment, a lifelong learning policy should be established and promoted by the government. Policies and public finance expenditure to support employment, reemployment, and working conditions modification for older adults are also needed. Secondly, negative correlations between mental health and NLTSS remind us of the need to improve the elderly’s mental health. As one of the important parts of successful aging [63], mental health could be improved by increasing the integrity of the elderly [64], and integrity could be achieved through continued personal growth, the development of satisfaction, or having purpose in life [65]. According to a past study, seniors who accept themselves, have good family relationships, and embrace the concept that they are never too old to learn are more likely to experience positive mental health [66].

### 4.3. Limitations

There are some limitations to the present study that should be addressed. Firstly, one indicator for the latent variable of NLTSS in the SEM hypothesis model, namely NLTSS in this study, was a nominal variable. However, the type of variable typically used for SEM analysis is ordinal variables. To date, a national LTSS system has not been established in China, which might include such elements as an assessment of different levels of NLTSS or an LTSS refunding system. This made the evaluation of the NLTSS more difficult than it would be for the research conducted in countries that have founded LTC insurance or LTSS programs, such as Japan, South Korea, Germany, and the USA Different levels of NLTSS have been clearly given by the government in the above countries, yet in the current study, we chose to assess the NLTSS of the subjects by asking them ‘Do you need LTSS’, with the answers given as ‘Yes/No’. Further discussion on the assessment of NLTSS by self-report versus functional assessment by doctors is needed. Secondly, indicators for mental health in this study are ‘life satisfaction’, ‘subjective health’, and ‘mental health status in the last month’. These questions mainly focus on cognitions, yet information about mental health problems, such as the conditions of depression, stress, schizophrenia, or other mental disorders of the subjects, were not included in the present study due to time limitations, financial support, and content of the questionnaire.

### 4.4. Future Research

The effect of SES on mental health and NLTSS of Chinese elderly was examined in this study. In turn, the status of mental health and NLTSS of the elderly may also affect their SES. A future study is needed to clarify the effect from mental health and NLTSS on SES using SEM. Moreover, according to the WHO’s definition of health, besides mental health, there are also physical health and social health. More research is needed to examine the relationship of these types of health with SES and NLTSS. Matching studies of home and community-based services to level of functional ability with long-term care facilities reserved for those with the highest disability or a lack of informal caregiver support at home are also needed in the future, to explore preferences for type of care by the elderly.

## 5. Conclusions

The current study clarified the empirical association between SES, mental health, and NLTSS among Chinese elderly in Shandong Province. Mental health was found to be significantly and negatively associated with the NLTSS for both male elderly and female elderly, with a slightly stronger effect among the male elderly. A significant and negative relationship was observed between SES and NLTSS for both genders, while the association was stronger among the female elderly. SES exerted a positive effect on mental health for both male elderly and female elderly, and a slightly stronger effect was found among the male elderly.

## Figures and Tables

**Table 1:** Measurements of the variables in this study.

Variables	Definition	Measurements	Options
Socioeconomic Status	A broad concept that compositely assess an individual’s economic and sociological position in relation to others.	Education	(1) Illiteracy
			(2) Graduate from Elementary School
			(3) Graduate from Junior Middle School
			(4) Graduate from Senior Middle School and above
		Annual income	(1) less than 2200 RMB (USD = 326)
			(2) from 2201 to 3300 RMB (USD = 489)
			(3) from 3301 to 6200 RMB (USD = 919)
			(4) from 6201 to 21,400 RMB (USD = 3170)
			(5) more than 21,400 RMB (USD = 3170)
		Job before retire	(1) Professionals/technical
			(2) Leaders of government/public institution
			(3) Clerks or staff
			(4) Businessman/commercial servants
			(5) Farming/forestry/fishing industry
			(6) Transportation industry/manual workers
			(7) Privately/individually owned business
			(8) Other jobs.
Mental Health	A state of well-being in which every individual realizes his or her own potential, can cope with the normal stresses of life, can work productively and fruitfully, and is able to make a contribution to her or his community.	How do you feel about your health status (subjective health)?	(1) Very bad
			(2) Fairly bad
			(3) Moderately
			(4) Fairly good
			(5) Very good
		How about your mental health status last month (mental health last month)?	(1) Very bad
			(2) Bad
			(3) Moderately
			(4) Good
			(5) Very good
		Are you satisfied with your life (life satisfaction)?	(1) Very Dissatisfied
			(2) Fairly dissatisfied
			(3) Moderately
			(4) Fairly satisfied
			(5) Very satisfied
Need for long-term services and supports	The subjectively assessment of need for long-term services and supports by the elderly, which include both home and community-based services, as well as long-term care facilities.	long-term services and supports needs	(1) Not needed
			(2) Needed
		ADL Score	(1) 14
			(2) 15
			(3) 16
			(4) 17
			(5) 18–27
			(6) ≥28

ADL: activities of daily living.

**Table 2:** Characteristic of Participants by LTSSN.

Variable	LTSSN						χ^2^ Test
	No Need (*n* = 6768)		Need (*n* = 302)		Total (*n* = 7070)		
	*n*	%	*n*	%	*n*	%	
Gender							
Male	2722	95.6%	124	4.4%	2846	100.0%	0.085 *p* = 0.771
Female	4046	95.8%	178	4.2%	4224	100.0%	
Age							
60–64	1520	96.4%	57	3.6%	1577	100.0%	31.767 *p* < 0.001
65–69	2060	96.8%	69	3.2%	2129	100.0%	
70–74	1711	96.1%	69	3.9%	1780	100.0%	
>75	1477	93.2%	107	6.8%	1584	100.0%	

LTSSN = long-term services and supports needs.

**Table 3:** Descriptive SES (socioeconomic status) Characteristic of Participants by Gender.

Variable	Gender						χ^2^ Test
	Male (*n* = 2846)		Female (*n* = 4224)		Total (*n* = 7070)		
	*n*	%	*n*	%	*n*	%	
Education (graduation from)							
Illiteracy	491	21.6%	1779	78.4%	2270	100.0%	561.142 *p* < 0.001
Elementary School	1294	44.3%	1630	55.7%	2924	100.0%	
Junior Middle School	718	54.6%	597	45.4%	1315	100.0%	
Senior Middle School and above	343	61.1%	218	38.9%	561	100.0%	
Income (RMB per year)							
0–2200	505	30.0%	1180	70.0%	1685	100.0%	117.736 *p* < 0.001
2201–3300	439	38.1%	714	61.9%	1153	100.0%	
3301–6200	607	43.2%	797	56.8%	1404	100.0%	
6201–21,400	652	46.1%	762	53.9%	1414	100.0%	
>21,400	643	45.5%	771	54.5%	1414	100.0%	
Job Before Retire							
Professionals/technical	243	55.0%	199	45.0%	442	100.0%	121.792 *p* < 0.001
Leaders of government/public institution	111	61.7%	69	38.3%	180	100.0%	
Clerks or staff	87	53.0%	77	47.0%	164	100.0%	
Businessman/commercial servants	28	24.1%	88	75.9%	116	100.0%	
Farming/ forestry/fishing	2107	38.5%	3372	61.5%	5479	100.0%	
Transportation industry/manual workers	174	45.4%	209	54.6%	383	100.0%	
Privately/individually-owned business	14	43.8%	18	56.3%	32	100.0%	
Other jobs	82	29.9%	192	70.1%	274	100.0%	

**Table 4:** Descriptive Mental Health and NLTSS (the need for long-term services and support) Characteristic of Participants by Gender.

Variable	Gender						χ^2^ Test
	Male (*n* = 2846)		Female (*n* = 4224)		Total (*n* = 7070)		
	*n*	%	*n*	%	*n*	%	
**Subjective Health**							
Very bad	55	43.3%	72	56.7%	127	100.0%	23.336 *p* < 0.001
Fairly bad	434	37.1%	735	62.9%	1169	100.0%	
Moderately	747	37.5%	1245	62.5%	1992	100.0%	
Fairly good	1098	41.6%	1541	58.4%	2639	100.0%	
Very good	512	44.8%	631	55.2%	1143	100.0%	
**Life Satisfaction**							
Very Dissatisfied	33	37.9%	54	62.1%	87	100.0%	5.400 *p* = 0.249
Fairly dissatisfied	35	39.8%	53	60.2%	88	100.0%	
Moderately	47	34.8%	88	65.2%	135	100.0%	
Fairly satisfied	1103	39.1%	1719	60.9%	2822	100.0%	
Very satisfied	1628	41.3%	2310	58.7%	3938	100.0%	
**Mental Health Last Month**							
Very bad	18	46.2%	21	53.8%	39	100.0%	25.614 *p* < 0.001
Bad	107	37.2%	181	62.8%	288	100.0%	
Moderately	418	35.1%	774	64.9%	1192	100.0%	
Good	1347	40.1%	2014	59.9%	3361	100.0%	
Very good	956	43.7%	1234	56.3%	2190	100.0%	
**LTSSN**							
Not needed	2722	40.2%	4046	59.8%	6768	100.0%	0.085 *p* = 0.771
Needed	124	41.1%	178	58.9%	302	100.0%	
**ADL Score**							
14	2274	41.6%	3193	58.4%	5467	100.0%	25.090 *p* < 0.001
15	171	35.5%	311	64.5%	482	100.0%	
16	67	30.2%	155	69.8%	222	100.0%	
17	70	34.1%	135	65.9%	205	100.0%	
18–27	191	36.4%	334	63.6%	525	100.0%	
≥28	73	43.2%	96	56.8%	169	100.0%	

LTSSN = Long-term Services and Supports, ADL = Activities of Daily Life.

**Table 5:** Multi-group Model Invariance Test.

Model	χ^2^	*df*	χ^2^/*df*	GFI	AGFI	CFI	RMSEA	ΔCFI	ΔRMSEA
M_1_	209.672 ***	30	6.989	0.993	0.983	0.975	0.029	---	---
M_2_	209.672 ***	30	6.989	0.993	0.983	0.975	0.029	0	0
M_3_	209.672 ***	30	6.989	0.993	0.983	0.975	0.029	0	0
M_4_	275.631 ***	35	7.875	0.991	0.980	0.966	0.031	0.009	0.002
M_5_	290.786 ***	38	7.652	0.990	0.981	0.965	0.031	0.001	0

M_1_ = Male Elderly; M_2_ = Female Elderly; M_3_ = Unconstrained; M_4_ = Measurement Weights; M_5_ = Structural Weights. *** *p* < 0.001. χ^2^ = Chi square; *df* =degrees of freedom; GFI = Goodness of Fit Index; AGFI = Adjusted Goodness of Fit Index; CFI = Comparative Fitness Index; RMSEA = Root-mean Square Error of Approximation ΔCFI = change of CFI; ΔRMSEA = change of RMSEA.

**Table 6:** Standardized Effects between Socioeconomic Status, Mental Health and NLTSS by Gender.

Variable	Direct		Indirect		Total	
	Male	Female	Male	Female	Male	Female
Socioeconomic Status→NLTSS	−0.117 ***	−0.252 ***	−0.113 ***	−0.064 ***	−0.230 ***	−0.316 ***
Mental Health→NLTSS	−0.400 ***	−0.300 ***	-	-	−0.400 ***	−0.300 ***
Socioeconomic Status→Mental Health	0.281 ***	0.214 ***	-	-	0.281 ***	0.214 ***

NLTSS = Need for Long-term Services and Supports, *** *p* < 0.001.

## Abbreviations

SES	socioeconomic status
NLTSS	need for long-term services and support
LTCI	long-term care insurance
SEM	structural equation modeling
GDP	gross domestic product
PSUs	primary sampling units
SSUs	secondary sampling units
ADL	activities of daily living
IADL	instrumental activities of daily living
PSMS	physical self-maintenance scale
SPSS	statistical package for social science
LTSSN	long-term services and support needs
GFI	goodness of fit index
AGFI	adjusted goodness of fit index
CFI	comparative fit index
RMSEA	root mean square error of approximation
UEBMI	urban employee basic medical insurance
URBMI	urban resident basic medical insurance
NRCMS	new rural cooperative medical System

## Appendix A. Brief Introduction to SEM

SEM is a collection of statistical techniques which examine the relationships between one or more independent variables and one or more dependent variables, and determine the extent to which the theoretical model is supported by the empirical data. Both the independent variables and dependent variables could be either continuous or discrete. Variables are represented by circles or ovals in path diagrams of SEM, and the relationships between variables are indicated by lines. The lack of a line connecting variables implies that no direct relationship has been hypothesized [67].

Several terms are related to SEM. The observed, measured, or indicator variables are a set of variables that we use to define or infer the latent variable or construct. Latent variables (constructs or factors) are variables that are not directly observable or measured, which could be inferred from a set of variables that we used in tests, surveys, and so on [68].

## Appendix B. Model Fitness Index of SEM

Assessment of the model fitness calculates how the theoretical model might be consistent with the empirical data. The chi-square test, GFI, AGFI, CFI, and RMSEA were reported for analysis of model fitness, as in many previous studies. When sample sizes are large (as in the present study), a non-significant chi-square is rarely obtained [69,70], and this test is too dependent on sample size to be useful in many situations [71]. The chi-square test was thus not used to compute for fitness statistics in this study, although it was shown. GFI, AGFI, CFI, and RMSEA were instead adopted as the fitness indices in this study. The hypothetical models are regarded to be well fitted to the data when *p* > 0.05; GFI > 0.90, AGFI > 0.90, CFI > 0.90, and RMSEA < 0.05 [72].

## Appendix C. Measurement Invariance across Groups in SEM

Before the discussion of comparison of gender differences in the structural model of SEM, the multi-group model invariance should be tested. If the test was passed and measurement invariance across groups was established, the comparison of gender differences could follow. The partial measurement invariance was employed to test the measurement invariance over multi-groups models by SEM in this study, which was able to assess the invariance by the change of CFI (ΔCFI) and the change of RMSEA (ΔRMSEA) for the comparison of a less restricted model with a more constrained model. The basic test strategy is to outline the response to model trimming, where an initial unconstrained model is gradually restricted by adding constraints [73].

ΔCFI is independent of both model complexity and sample size, and not correlated with the overall fit measurements. A change of <0.010 in CFI suggests the null hypothesis of no difference should be accepted, indicating that measurement invariance across groups was established [74]. For the ΔRMSEA, when the sample size is more than 300, a change of <0.015 in RMSEA (ΔRMSEA) indicates that measurement invariance across groups was established [75].
